# Computational Modeling for Cardiac Resynchronization Therapy

**DOI:** 10.1007/s12265-017-9779-4

**Published:** 2018-01-11

**Authors:** Angela W C Lee, Caroline Mendonca Costa, Marina Strocchi, Christopher A Rinaldi, Steven A Niederer

**Affiliations:** 1 School of Biomedical Engineering and Imaging Sciences, King’s College London, London, UK; 2 Department of Cardiology, Guy’s and St. Thomas’ Hospital, London, UK

**Keywords:** Cardiac resynchronisation therapy, Left bundle branch block, Electrophysiology modeling, Electromechanical modeling, Hemodynamic modeling, Computer-based model

## Abstract

Cardiac resynchronization therapy (CRT) is an effective treatment for heart failure (HF) patients with an electrical substrate pathology causing ventricular dyssynchrony. However 40–50% of patients do not respond to treatment. Cardiac modeling of the electrophysiology, electromechanics, and hemodynamics of the heart has been used to study mechanisms behind HF pathology and CRT response. Recently, multi-scale dyssynchronous HF models have been used to study optimal device settings and optimal lead locations, investigate the underlying cardiac pathophysiology, as well as investigate emerging technologies proposed to treat cardiac dyssynchrony. However the breadth of patient and experimental data required to create and parameterize these models and the computational resources required currently limits the use of these models to small patient numbers. In the future, once these technical challenges are overcome, biophysically based models of the heart have the potential to become a clinical tool to aid in the diagnosis and treatment of HF.

## Introduction

Heart failure (HF) is a complex and varied disease which results from structural or functional abnormalities impairing the ability of the heart to pump blood and deliver sufficient oxygen to meet the body’s metabolic needs. Pathological remodeling occurs with the progression of HF and refers to the macroscale changes in the mass and shape of the heart as well as the microscale changes in the myocyte cellular structure and ionic channel distribution and density which detrimentally affect cardiac function over time. Conceptually “reverse remodeling” is the reversal of this progressive deterioration, and is indicated with surrogate markers such as a reduction in ventricular volume and mass, as well as improvements in the contractile function of the heart. Reverse remodeling has been shown to be correlated with better therapeutic outcomes for patients [1].

Large clinical trials (REVERSE, MADIT-CRT, and RAFT) have found cardiac resynchronization therapy (CRT) to be an effective treatment which possibly promotes reverse remodeling for patients with drug refractory mild to severe HF with prolonged QRS duration [2–4]. Prolongation of the QRS duration is a sign of abnormal electrical activation across the ventricles, such as with the left bundle branch block (LBBB), leading to mechanical dyssynchrony in the contraction of the heart. In a normal heartbeat, the electrical activation spreads throughout the atria, passes slowly through the AV node, before rapidly spreading through the bundle of His, down though the right and left bundle branches and into the Purkinje fibers to activate the normal myocardium. In LBBB, as per the name, an electrical blockage occurs in the left bundle branch, causing a dyssynchronous electrical activation of the ventricles. In CRT, the heart is artificially paced in the right atrium, right ventricle (RV), and left ventricle (LV) to resynchronize the electrical activation and mechanical contraction of the heart. Device settings allow for clinicians to set the time delay between activation at the atria and then subsequently the ventricles (AVD) and the time delay between the ventricles (VVD), with a positive VVD indicating RV-first pacing.

Multicenter studies have shown that CRT reduces mortality and morbidity and reduces HF hospitalizations [4]. Clinical studies have observed that the ventricular function improves both acutely and over the longer term with CRT, with the latter possibly due to reverse remodeling [5]. Despite the benefits of CRT, within the cohort of HF patients that are indicated for implant, there remains 40–50% of patients that do not respond positively to treatment [6, 7]. This lack of response to treatment has been posited to be due to suboptimal lead location or suboptimal device settings (AVD/VVD) or whether the patients underlying pathology was amenable to CRT. Previous studies have found that in a large proportion of non-responders, the device settings or the lead positions were suboptimal [8]. Computer models of the heart have thus been used to identify the optimal lead location and pacing settings of the heart to predict the response of the heart to CRT, as well as to better understand the underlying pathologies that give rise to cardiac dyssynchrony.

The goal of CRT is to stop or reverse the progression of HF by resynchronizing the electrical activation and mechanical contraction of the ventricles of the heart leading to a functional improvement in the pumping of blood throughout the circulatory system. Computer models can be used to simulate the electrical activity on the heart from the cellular level through to the tissue level (electrophysiology models), giving rise to contraction of the ventricles (mechanical models), as well as the pumping of the blood throughout the cardiac system (circulatory models).

**Fig. 1:**
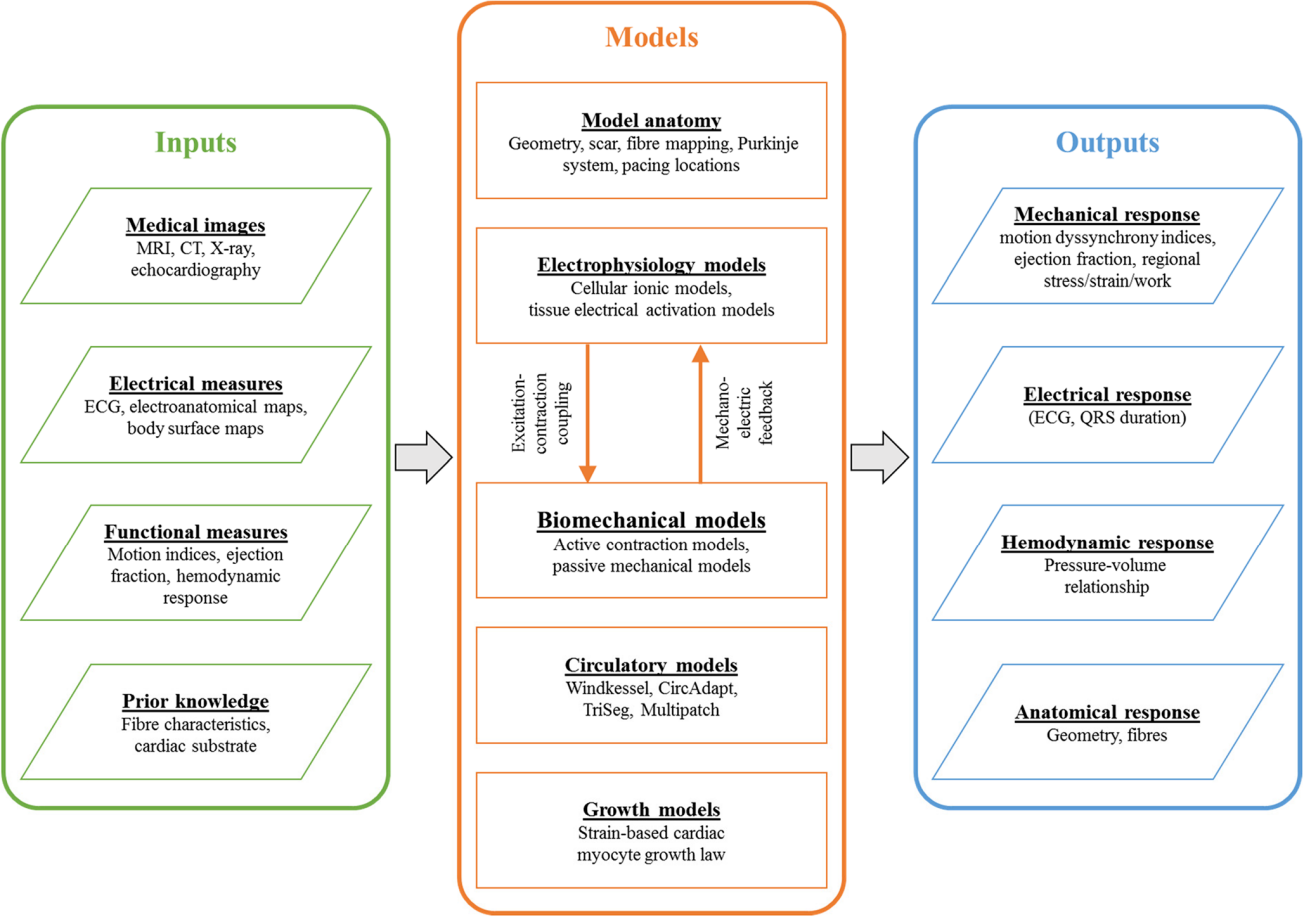
Schematic of the modeling pipeline for biophysical simulations of LBBB and CRT response. Patient data and prior knowledge can be used to create and parameterize the anatomical, electrophysiology, biomechanical, circulatory, or growth models of the heart. The model outputs can be used to infer and integrate information regarding the diseased heart, generate hypotheses, and predict responses to LBBB and CRT. Current state-of-the-art models do not encompass this entire theoretical framework, but rather only aspects of it depending on the availability of data, computational resources, and clinical question of interest

A schematic of how these models relate to each other is shown in Fig. 1. Medical images and prior knowledge are used as inputs to generate anatomical models of the heart that are patient or subject specific. Patient-specific anatomical models are then used to study the electrophysiology and electromechanics of the heart. Electrical measurements are used to parameterize and validate the electrophysiology models of the heart. Functional measurements are used as inputs to the circulatory and mechanical models of the heart. Prior knowledge from literature is used to constrain the models to physiologically plausible conditions. The outcomes from the models include changes the mechanical, electrical, hemodynamic, and anatomical responses to LBBB and CRT. In this review, we will focus on the contribution of electrophysiology, mechanics, and circulatory computer models of the heart to understanding LBBB and CRT response.

## Modeling Anatomy

### Geometry

The first electromechanical model investigating LBBB and CRT was based on a generic idealized shape of the heart [9]. Though generic shape models are still useful in providing mechanistic insights into the underlying substrate [10], because of the importance of scar location [11] and burden [12, 13], pacing settings and pacing location [14], the general trend in computer models of LBBB and CRT has been towards personalized shape models. Segmentations of non-invasive medical imaging techniques (CT, MR, X-ray, and echo) of the heart provide the anatomical information required to build subject-specific canine models [15, 16] or patient-specific models [17–20]. Computational meshes can then be built from segmentations of the heart models for electrophysiological and mechanical simulations. Patient-specific anatomical meshes used in electrophysiology and electromechanical simulations of the heart have focused on the electrical activation and mechanical deformation of the ventricles. The other cardiac structures are typically ignored in these models. In future studies, the importance of the atria and other cardiac structures such as the pericardium, valves as well as the blood vessels in simulating CRT and LBBB will need to be determined.

### Fibers

Myofiber orientation plays an important role in electrical and mechanical properties of cardiac tissue. The electrical activation in the heart spreads two- to sevenfold faster along the fiber rather than transverse to the fiber direction [21–23]. Furthermore, biaxial tests on cubic samples of mammal myocardium found that cardiac tissue has an increased stiffness in the fiber direction [24], while active contraction acts predominantly in the fiber direction. For these reasons, a physiological representation of local myofiber orientation throughout the myocardium is fundamental.

A large number of studies of heart biomechanics [9, 10, 18–20, 25–32] have used rule-based methods [26] based on measurements of the fiber angles [33–35] to define the fiber orientation. Recently, there has been increasing interest in personalizing the fiber orientation of the heart using in vivo [36–42] or ex vivo diffusion tensor MRI (DT-MRI) [34, 43, 44].

Currently, in vivo DT-MRI has been performed on healthy volunteers’ scans [40, 45] and patients with hypertrophic cardiomyopathy [46] with a maximum of three high-resolution image slices acquired after multiple breath holds. Though DT-MRI is a promising technology for personalizing the fiber orientation, the clinical translation of DT-MRI remains elusive. Until such technical challenges regarding the signal-to-noise ratio, long acquisition times requiring multiple breath holds, and the bulk motion of the heart obscuring the fiber structure measurements are solved, imaging the whole heart and incorporating patient-specific fiber orientations into computer models of the heart is unfeasible. The spread of the electrical activity with rule-based fiber orientation and those derived from DT-MRI in rat [47] and canine models [26] have also been found to be comparable, justifying the continuing use of rule-based methods.

### Scar

The importance of scar in response to CRT has been consistently observed in clinical studies, where pacing within scar regions has a detrimental effect on the response to CRT [48]. In addition, the total scar burden has been found to correlate to the response for CRT [49]. In scar tissue, the fiber orientations have been found to be disordered with a higher mechanical stiffness and reduced electrical conductivity [50, 51]. This has been represented in models of HF and LBBB, with reduced electrical conductivity, less anisotropic material laws, and increased passive stiffness values [25, 27], and changes in the active tension models to reflect the contraction force in scarred regions [12]. Computer models of CRT have found that, excluding infarcted regions as prospective pacing sites, the optimal pacing site depends based on the location of the scar [11].

In the clinical setting, scar regions are typically segmented from contrast-enhanced cardiac MRI using signal intensity thresholding techniques. Typically, the ventricular wall is manually or automatically delineated and the signal intensity within the wall is used to define regions of scar. The most widely used methods are the full-width-half-max [52] and standard deviation [53] methods. In the former, image regions with signal intensity larger than the 50% of the maximum intensity within the wall are selected as scar. In the latter, a region of healthy myocardium is manually selected, the mean and standard deviation of the signal intensities within this region are computed and scar is defined as the region with signal intensity above the mean healthy intensity plus 2 or 3 standard deviations. Although there is currently no consensus on which scar segmentation technique should be used [54], the full-width-half-max model has been shown to be more reproducible [55], as it does not require manual selection of a region of healthy myocardium.

Different scar segmentation methods yield differences in scar size, shape, and distribution [54], which could affect CRT modeling studies. Scar location plays a significant role in pacing site choice, as pacing adjacent to a scar has been shown to yield poor CRT response [56]. Thus, inaccurate scar identification could affect results when using models to identify optimal pacing sites. However, to the best of our knowledge, a comparison of simulation outcomes in this scenario using different scar segmentation methods has not yet been done.

**Fig. 2:**
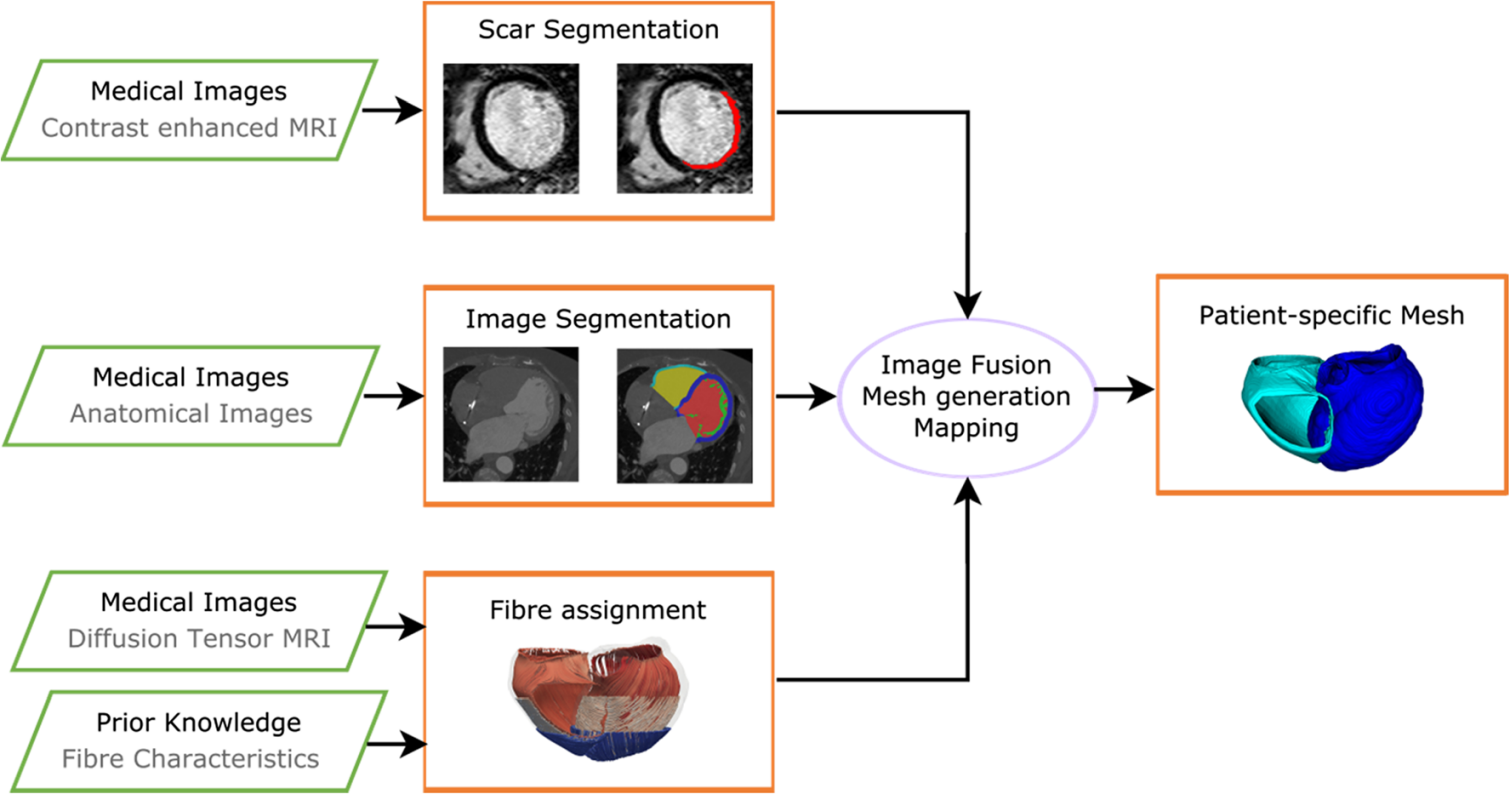
A schematic of a workflow for the generation of patient-specific anatomical model of the heart from medical images. Segmentations of the anatomical image are used to generate patient-specific meshes. Scar segmentations (from contrast-enhanced MR) and fiber information (from rule-based or image-based methods) can then be mapped onto the mesh

An outline of typical workflow for generating the anatomical models used in electrophysiology and electromechanical cardiac simulations is shown in Fig. 2. Image segmentation of the anatomical medical images (such as from MR or CT) identifies the patient-specific geometry of the ventricles, from which anatomical meshes for electrophysiology or mechanical modeling are generated. The locations of scar tissue can be segmented from contrast-enhanced MRI. Image registration of the contrast-enhanced MR with the anatomical image allows the scar segmentations to be mapped onto the anatomical mesh. The fiber orientations in the ventricles can then be assigned from rule-based methods derived from prior knowledge or potentially from diffusion tensor imaging.

## Clinical Measurements

### Electrical Measurements

Computer models need to reflect the changes in the electrical activation pattern that HF or CRT induces. In the typical clinical setting, electrical dyssynchrony in the heart is measured using 12-lead electrocardiograms (ECG). The duration and morphology of the QRS from the 12-lead ECG are used as guidelines for patient selection for CRT. However, the limitation of this widely used clinical tool is that the small number of leads recording at a distance from the heart may not fully capture the electrical activation of the heart. In computer models, the QRS duration is often used as a surrogate for time taken for the electrical activation to pass through the ventricles to parameterize the conduction velocity [20, 28–30, 57–59].

A more comprehensive picture of the electrical activation across the torso can be achieved with non-invasive body surface potential mapping (BSPM). BSPM captures many more ECGs across the torso via a multi-electrode vest (with commercial versions recording up to 256 ECGs). Though the diagnostic value of BSPM can be larger than that of 12-lead ECG [60, 61], the difficulty of interpretation of BSPM and limited access means that BSPM has yet to be adopted into widespread clinical use for CRT patients. Invasive electro-anatomical maps (EAM) of the LV endocardium and the coronary veins provide more accurate and direct measurements of changing electrical patterns in the ventricles. However, the invasive nature of these measurements mean that this data can only be collected intra-procedurally and thus, the progression of the changes in the heart in response to CRT is not tracked using these measurements. To date, only one research group has used BSPM to parameterize electrophysiology models of the heart [62], while mostly the QRS duration and/or EAM have been used to parameterize and validate cardiac computer models [18, 20, 28, 29, 57–59, 63].

### Functional Measurements

Computer models also need to reflect the functional changes in the heart due to LBBB and CRT response. Non-invasive imaging (Cine MRI or echocardiography) can capture the deformation of the heart throughout the cardiac cycle. Clinical measures such as LV volume transient (absolute and regional), end-diastolic volume, end-systolic volume, ejection fraction (calculated as the percent of blood ejected from the ventricles), LV mass, and LV motion dyssynchrony indices can be derived from these images. Wall motion measurements from tagged MRI can be used to validate deformations predicted by the model [28, 64].

Pressure measurements can be both invasive (pressure catheters measuring the change in LV pressure intra-procedurally) or non-invasive (via arterial pressure measurements). The pressure and/or volume transients are used to adapt the geometry parameters (in the circulatory models [64–66]) and the active and passive material parameters (in the biomechanical models [18, 20, 27–30, 57] of the heart.

## Electrophysiology Models

**Fig. 3:**
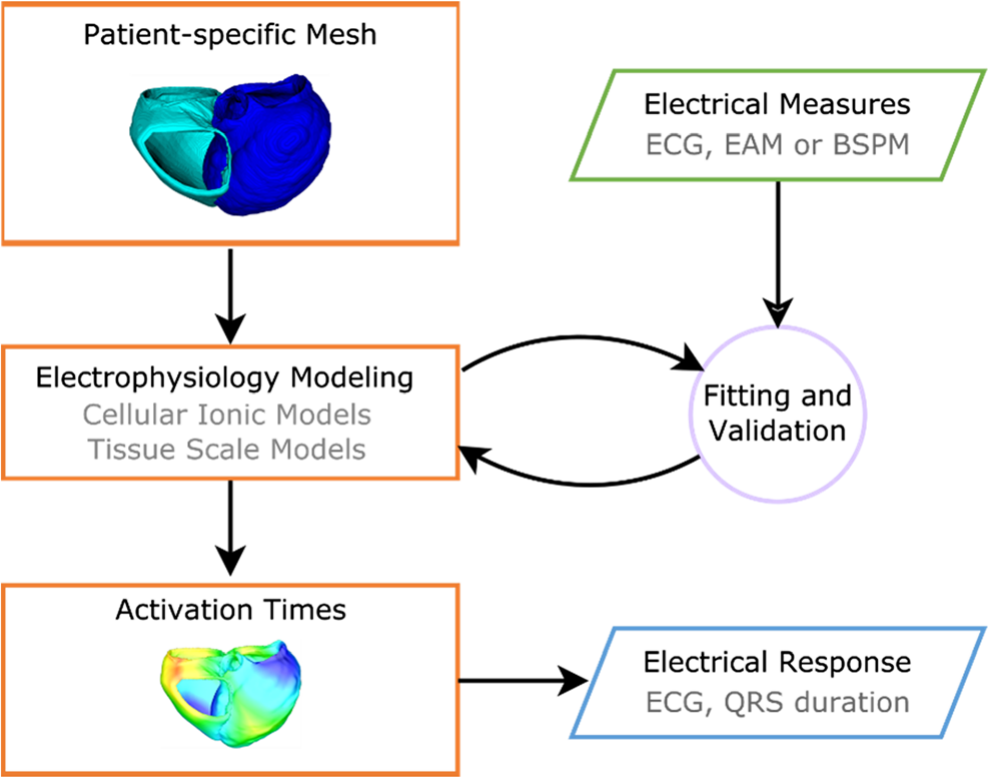
A schematic representation of the electrophysiology models is shown. Personalized shape models of the heart and electrical measures, such as the ECG, Electro-anatomical Maps (EAM), or Body Surface Potential Maps (BSPM) are used as inputs into multi-scale electrophysiology models of the heart to simulate the electrical activation times across the ventricles. The model outcomes can be evaluated in terms of clinical measures such as ECG traces and QRS duration

In the section “Modeling Geometry,” we described a typical workflow to generate geometrical models of the heart based on patient-specific anatomy. Such patient-specific anatomical models may be combined with electrophysiological models to simulate activation sequences and study a patient’s electrical response to CRT. To accurately simulate a patient’s activation sequence, model parameters must be fitted to electrical measurements, such as ECG, EAM, and BSPM, and validated. A typical workflow for electrophysiology personalization is shown in Fig. 3. Different types of electrophysiology models have been employed in CRT studies, such as the Monodomain and Bidomain models, the Eikonal model, Cellular Automaton models, and Surface Source models.

The monodomain and bidomain models belong to the class of multi-scale models of cardiac electrophysiology. Both models describe electrical activity at the cellular scale, where action potentials are generated, and at the tissue scale, where action potentials propagate from one cell to another. While the Monodomain model only describes propagation in the intracellular space, the Bidomain model also includes the extracellular domain. The Bidomain model is the more complete electrophysiology model of the two and as such, also more computationally expensive. Thus, most CRT studies employ the Monodomain model [10, 32, 58, 67].

Although the monodomain model is computationally cheaper than the bidomain model, solving the monodomain model still requires significant computational effort to resolve the steep wave-front of activation. To overcome this limitation, the Eikonal model has also been used in CRT modeling studies [68] to simulate activation sequences. This model describes the location of the activation upstroke without resolving the complex action potential kinetics, rendering it computationally very efficient.

With a similar goal, cellular automaton models [69] have also been employed in recent CRT studies [70–73]. In this model, instead of explicitly solving the complex current flow interaction between the intracellular and extracellular domains, action potentials are pre-calculated based on ionic current equations. These are then stored and applied as a set of rules allowing fast computation of ventricular activation and repolarization sequences.

A type of surface source model called the Equivalent Dipole Layer model, which computes body surface potentials from the transmembrane potential on the myocardial surface (source) based on the Bidomain formulation [74, 75], has also been employed in CRT studies [76].

At the organ scale, the cardiac bioelectric behavior is controlled by a cardiac conduction system. In the ventricles, this system is typically referred to as the Purkinje system (PS), and is composed of the bundle of His, the left and right bundle branches, and an extensive Purkinje network, which connects to the myocardium at the Purkinje-ventricular junctions. Propagation in the PS is two to three times faster than in the myocardium, allowing fast distribution of impulses over the endocardium and ensuring ventricular synchrony. Thus, when modeling the activation sequence at the organ scale, it is important to include the PS properties into the model, as it significantly affects the activation pattern in the heart.

In CRT models of realistic ventricular anatomy, the PS has been modeled as a tree of one-dimensional elements coupled to the myocardium [32, 68] or as a fast conducting endocardial layer, which approximates the PS by assigning tissue conductivities to the endocardium that match the conduction velocity in the PS [10, 67]. Models with the PS represented as a tree of one-dimensional elements have been used to investigate the effect of AVD on the distribution of activation times in the myocardium during CRT and demonstrated that a 30-ms AVD yields an activation pattern more similar to healthy activation than a 0-ms AVD [68].

A similar approach was used to investigate the role of electrical conduction in the PS during CRT pacing in failing hearts [32]. Motivated by experimental evidence of retrograde activation in the PS [77, 78], Romero et al. [32] compared a realistic PS represented by a tree of one-dimensional elements with a fast endocardial layer. Their results show that retrograde conduction in the PS is key to accurately estimate VVD and that a fast endocardial layer cannot accurately capture this effect [32]. However, a fast endocardial layer was employed in a biventricular (BiV) model to study the effect of endocardial versus epicardial pacing during CRT and showed good agreement with experimental results [10]. Their results demonstrated that early access to fast-conducting endocardial tissue reduces ventricular activation time during endocardial pacing, providing a physiological explanation for the observed benefit of endocardial pacing compared with epicardial pacing [79, 80]. They also showed that patients with concentric anatomical remodeling are more likely to benefit from endocardial pacing than patients with eccentric remodeling.

Computer models have been used to study the effect of CRT on electrical activation in the presence of the ECG characteristic of LBBB [58], which can be caused by conduction block in the left branch of the His bundle due to damage to the His fibers or due to myocardial uncoupling caused by reduced expression of connexins (gap junctions) in its vicinity. Their results show that myocardium uncoupling can mimic LBBB and account for an LBBB ECG pattern. In addition, they showed that CRT improves ventricular activation in the presence of LBBB but not in the case of myocardium uncoupling mimicking LBBB.

Electrophysiology simulations have also been used to optimize lead location, AVD, and VVD during CRT. These models offer the opportunity to carry out non-invasive, automatic optimization of CRT in silico. Briefly, the approach consists of simulating electrical activation in a realistic BiV anatomy for different AVD and VVD as well as several different lead locations and minimizing the error between the obtained activation sequences for each case against a simulated physiological case to determine which combination of AVD, VVD, and lead location yields the best acute response to CRT [70–73, 81]. Such models have demonstrated that patient-specific optimization of lead location and AVD and VVD can improve CRT efficacy and impact treatment success [31, 70, 73] and that the use of body surface potential maps can further improve in-silico CRT optimization [82].

Although computer models have been useful to simulate complex electrophysiological properties during CRT, tuning these models to accurately simulate a patient’s ventricular electrophysiology is challenging due to the high number of parameters involved. Therefore, Sanchez et al. [67] investigated the role of myocardial properties in the activation sequence on the LV endocardial surface and the ECG morphology in HF patients. Specifically, they analyzed intracellular and extracellular tissue conductivities and cellular membrane ionic properties. Their results show that the QRS complex and LV activation times are mainly determined by the sodium current and tissue conductivities; that the T-wave is mainly modulated by the calcium and rectifier-potassium currents; and that the cell surface-to-volume ratio affects all three properties. Moreover, the effects of changes in tissue properties vary between ECG leads, whereas ionic changes entail similar effects in all ECG leads.

The ECGSIM approach was used by van Huysduynen et al. [76]. ECGSIM is an ECG simulation program based on the equivalent dipole layer model, as previously described. The model incorporates heart and thorax geometry based on MRI, conduction heterogeneity, and transmural dispersion of repolarization (TDR). The approach was used to investigate if BiV pacing in CRT increases transmural dispersion of repolarization in comparison with conventional right ventricular (RV) pacing. Their results show that both pacing strategies increase TDR compared with healthy intrinsic activation, but that TDR during BiV pacing is not significantly larger than during RV pacing. Thus, increased TDR does not explain possible proarrhythmic effects of CRT.

## Mechanical Models

**Fig. 4:**
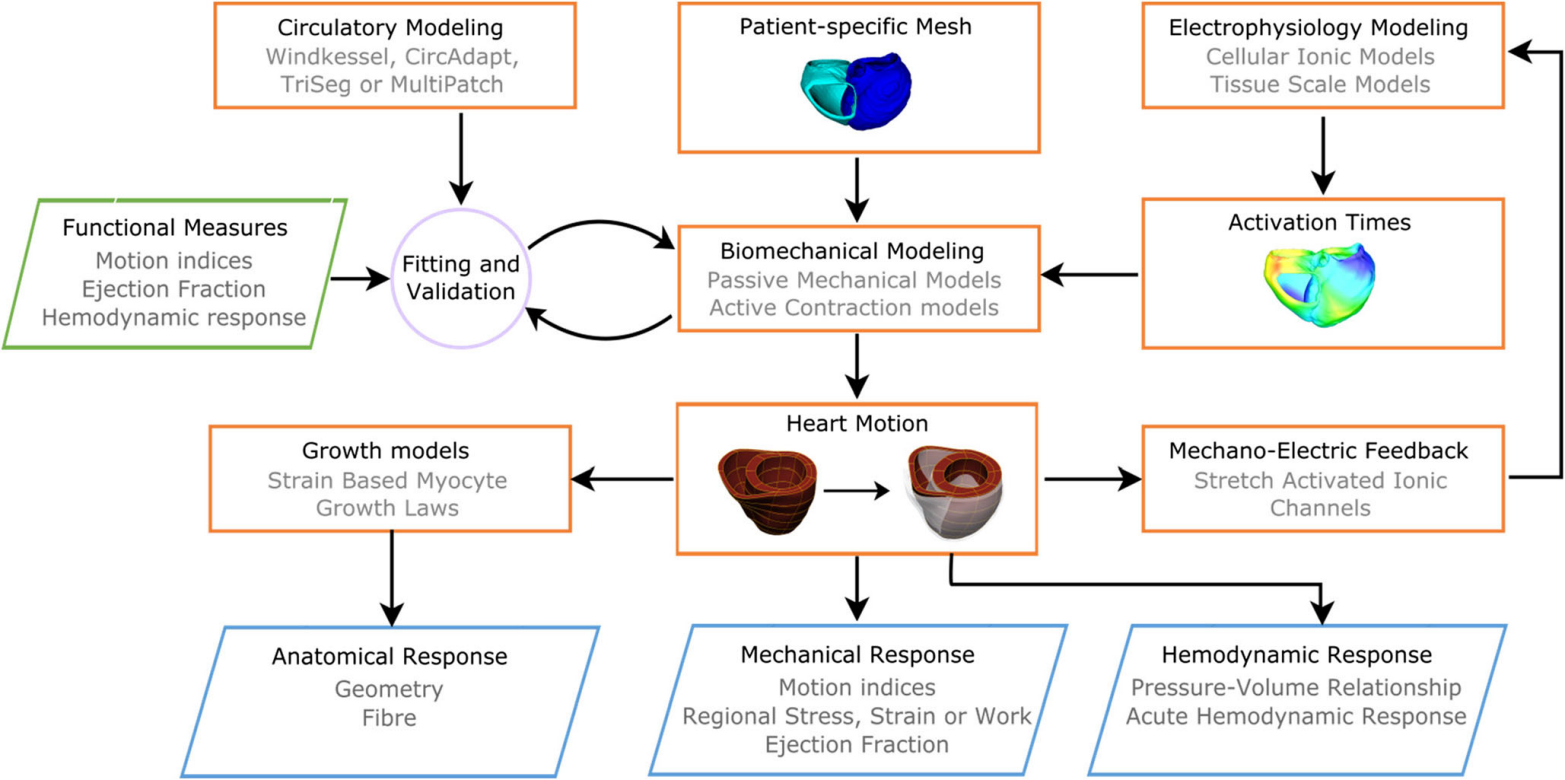
A schematic of representation of the electromechanical workflow is shown. Personalized shape models of the heart, electrophysiology models, and circulatory models are used as inputs into the biomechanical models of the heart which use finite elasticity equations to model dynamic cardiac deformations. Mechano-electric feedback via stretch activated ionic channels feedback into the electrophysiology modeling of the electrical activation time input into the mechanical model simulations. The model outcomes can be evaluated in terms of the mechanical and hemodynamic responses, and potentially the anatomical response (mediated with growth models)

Dyssynchrony in the electrical activation of the heart naturally leads to mechanical dyssynchrony in the heart. However, clinical studies have shown that resolving the electrical dyssynchrony does not necessarily improve the mechanical dyssynchrony [83–85]. For this reason, mechanical deformation throughout the cardiac cycle needs to be simulated as well. A typical workflow of electromechanics modeling of the heart is shown in Fig. 4. Generic or personalized shape models of the heart with mapped fiber orientation (from rule-based or image-based methods) and encompassing structural and functional heterogeneities (from regions such as scar) can be used to simulate the mechanical deformation of the heart using continuum mechanics equations solved in a Lagrangian reference frame [86].

To model the substantial deformation that the heart undergoes throughout the cardiac cycle, three components are required: a constitutive law for passive material properties of the myocardium, an active contraction model to represent active tension generation along myofibers, and a coupling method between the electrophysiology simulation and the mechanics simulation.

Passive material properties of the myocardium can be modeled using anisotropic (along the fiber, sheet, normal directions of the fiber field), hyperelastic (or viscoelastic) constitutive equations [87–89], or more typically using transversely isotropic [90, 91] material laws. In the context of patient-specific models, parameters of the constitutive equation are fitted and validated using functional clinical measurements, such as the passive pressure-volume relationship during ventricular filling [57].

The electrical activity throughout the myocardium leads to the mechanical contraction and relaxation of the heart through excitation-contraction coupling (ECC) [92]. On a cellular level, the depolarization of the myocyte gives rise to a Ca^2+^ signal that activates the sarcomeres, causing tension generation and contraction of the myocyte, which at the organ scale translates into pumping of blood in the heart. However, the mechanical contraction of heart can also effect the electrophysiology of the cardiac cells through mechano-electric feedback (MEF) [93], such as via stretch-activated ion channels [94, 95] to change the ionic currents in the cell.

Active tension generated along myofibers as a consequence of cellular excitation during systole is included in the mechanics model as an additional tension component in the fiber direction. There are three ways to model ECC in electromechanics models. The majority of electromechanical models of the heart have focused on *phenomenological coupling* [25, 96] by means of electrical activation time only and *weakly coupled* models [97, 98] by means of intracellular calcium transient. These two modeling strategies are based on the common assumption that the electrophysiology affects the mechanical contraction of the heart through ECC but any effect of the mechanical deformation on electrical propagation is negligible and can be ignored. In contrast, limited work being done with *strongly coupled* models which take into account the MEF [99, 100].

To date, computer models investigating CRT have done so with phenomenological and *weakly coupled* models, due to the large computational costs of implementing *strongly coupled* electromechanical models. In personalized models, parameters defining the active tension transient are fitted to match available clinical measurements for cardiac deformation during systole such as systolic pressure, volume transients, or cardiac motion from cine-MRI. The simulated mechanical deformation of the heart models can then be further analyzed to determine mechanical and hemodynamic response outcomes.

Most of the cardiac models studying LBBB and CRT response have neglected the longer-terms effect of pathological remodeling and reverse remodeling associated with the two states. Incorporation of growth models allows for simulations of the dynamic anatomical changes in the heart with the progression of LBBB or CRT response. In [101], the effects of myocyte shape changes in response to the strain on the cell were investigated on a canine model of LBBB. A later study by [102] investigated the effect of growth models on a human model with myocardial infarction. The general anatomical effects of pathological remodeling were captured in modeling studies (increase in LV mass, LV dilation, reduction in EF). However, cardiac remodeling encompasses changes to the electrics, mechanics, and function of the heart as well as volumetric changes. The effects of electrophysiology changes, alterations in the fiber orientations, and mechanical model material properties on pathological remodeling should also be investigated in future studies. Reversal of the growth model to capture the reverse remodeling effects associated with CRT still needs to be determined in order to model the chronic effects of CRT response.

Electromechanical models of the failing heart have been used to investigate optimizing the acute CRT response. One strategy for optimizing CRT is to maximize the acute hemodynamic response (AHR), given as the maximal rate of systolic left ventricular pressure rise [103, 104]. However, prior to achieving the lofty goal of translation of the biomechanical models of the heart to the CRT clinic, models need to prove their ability to accurately simulate clinical measurements.

In recent studies, patient-specific biomechanical models developed from extensive and rich clinical data were used to predict the acute hemodynamic effects of pacing protocols [20, 27, 29, 30, 105] and were able to achieve good agreement with hemodynamic measures. In these studies, small patient numbers were modeled (1–9 patients). This is in part due to the extensive data requirements (such as the invasive LV pressure recordings and electro-anatomical maps) and significant computational costs of creating and parameterizing personalized biophysically based cardiac models. To parameterize computer models, multiple simulations with different input parameters need to be run. Supercomputing resources allow for the simulation of cardiac electrophysiology at clinical time scales, allowing for the simulation of a single heart beat at 6.7 s [106] and 4 min [107]; however, this required computational resources on the order of 1.6 million cores and 16,384 cores, respectively. The large computational expense of computer models, requiring access to high performance computer facilities, limits the clinical usefulness of such methods.

The location of the LV pacing lead has been shown to have an effect on CRT response [108, 109], and suboptimal lead placement has been identified as a cause in 21% of CRT non-responders. Therefore, predicting the optimal LV pacing location is one of the goals of CRT electromechanical modeling. Constantino et al. [110] used a canine model, Pluijmert et al. [14] used a stylized human shape model, while patient-specific models were used in [28, 29] to identify the optimal LV pacing site. In these electromechanical modeling studies of CRT, the optimal LV pacing location based on maximizing AHR [14, 28, 29], maximizing stroke work [14], minimizing the electromechanical delay [110], or reducing the LV electrical activation time [28]) was found to be in the lateral LV free wall, which is broadly consistent with experimental canine [109, 111] and clinical studies [108, 112].

Optimizing the device settings (AVD/VVD) has been shown to improve the acute functional response, such as AHR and EF, to CRT in the clinical setting [113–117]. In contrast to the electrophysiological models discussed in the previous section, electromechanical models optimizing the AV/VV delay focus on measures of mechanical synchrony rather than electrical synchrony [29, 105]. Consistent with the literature [116, 118], electromechanical models have found that the optimal AV/VV delay is highly personalized [28, 31, 105] and that it changes with chronic CRT [29]. Although AV/VV delay optimization can improve response to CRT, in real-world practice, patients may be left with suboptimal device settings post-CRT due to lack of time and qualified staff [117, 119]. Computer models offer the potential to systemically evaluate optimal AV/VV delay settings outside the clinic.

In addition to using models to optimize CRT response to existing treatment methods, computer models of the heart have also been used to evaluate emerging technologies for delivering pacing to the heart [105]. Conventional CRT aims to resynchronize the heart by artificially pacing the heart in the RV and on the LV epicardium via the coronary sinus. Multisite pacing (MSP) stimulates multiple LV sites via leads placed in multiple CS tributary veins or with a multipolar LV pacing lead. Preliminary studies have shown MSP can improve CRT response in both the acute [120–125] and chronic time scales [126, 127]. Electromechanical models have shown that patients with ischemia have an improved response to MSP CRT in comparison to conventional CRT [105]. As ischemic patients are a subgroup of patients that have the poorest response to conventional CRT [128], MSP CRT is a promising new technology in improving response rates to CRT.

### Limitations of Electromechanical Models

Electromechanical models looking at optimizing the AV delay settings are ventricular models of the heart, neglecting the atria [29, 105]. In the normal heart, the time delay between the atria and ventricular electrical activation allows for atrial contribution to the ventricular preload. Recent work on canine heart modeling included the atrial contribution to the ventricular preload via alterations of a lumped parameter model of the circulatory system [129]. Though this model was able to account for the hemodynamic effects of the atria in AV optimization, the anatomical effects of the atria on the activation pattern is still disregarded. Four-chamber heart models [130], containing the atria and ventricles, have the potential to address this problem. The change in the atrial contribution with AV optimization is currently not evaluated in the human models and the consequence of neglecting the effects of the atria will need to be investigated in future studies.

Most published electromechanical models predicting CRT response have used the AHR as an outcome measure. Clinical studies have shown poor reproducibility of AHR due to biological variability, especially when only measured once [131]. In [131], it was suggested that repeated measurements (six or more) and relative measurements improves the reliability of AHR measurements. Increasingly, models have adopted the prediction of the more reliable relative measure of AHR; however, few clinical groups are making recordings with six repeats due to the inherent constraints in the clinic.

The predictability of AHR with regards to long-term therapeutic response is still controversial, with some studies finding no correlation to chronic remodeling (which is linked to better clinical outcomes) [132] and others finding a positive correlation to chronic remodeling [133]. Other measures of acute improvement such as diastolic parameters and pressure volume loop have also been proposed; however, they have yet to be linked to long-term clinical response [134, 135]. Despite clinical efforts to identify an acute response measure that predicts the long-term response to CRT, it remains elusive and continues to be a challenge for clinical research.

Ventricular models have tended to focus on the simulation of a single beat with boundary conditions being represented by Windkessel models. Contraction models connected to the circulatory models [110, 136, 137] have linked organ scale models to closed loop cardiovascular models. As the heart operates as two pumps in series, the blood leaving the right ventricle must equal the blood entering the left ventricle with each beat. To account for these hemodynamic effects requires modeling not only the ventricles but the closed loop circulatory system within which the heart operates. In a later study, additional growth models were further incorporated into the models described in [136] to investigate the long-term effects of LBBB [101]. The changes observed in the model were in line with experimental observations of the pathological remodeling effects of the progression of LBBB, such as LV dilation, reduction in LV ejection fraction, and occurrence of septal flash [138].

## Circulatory Models

The multi-scale approach of three-dimensional computational electromechanics models offers a highly detailed simulation of microscopic and macroscopic phenomena. However, clinical translation is limited by the large computational efforts required to solve the model, especially when dealing with electromechanical coupling. High computational costs also restrict the simulation to cardiac chambers and often the simulation of a single beat, even though ventricular filling from the pulmonary and systemic venous systems through the atria and resistance to ejection through the aorta and pulmonary artery are likely to play a role in CRT response and optimization.

An alternative model strategy is to discard or significantly reduce the representation of the patient anatomy and employ a zero-dimensional or reduced dimensional approach. This dimensionality reduction allows for much faster simulations (from several hours to seconds or minutes), therefore allowing additional components to be included in the model, such as the systemic and the pulmonary circulation, and to simulate the circulatory system as a closed loop over multiple beats [139–141].

**Fig. 5:**
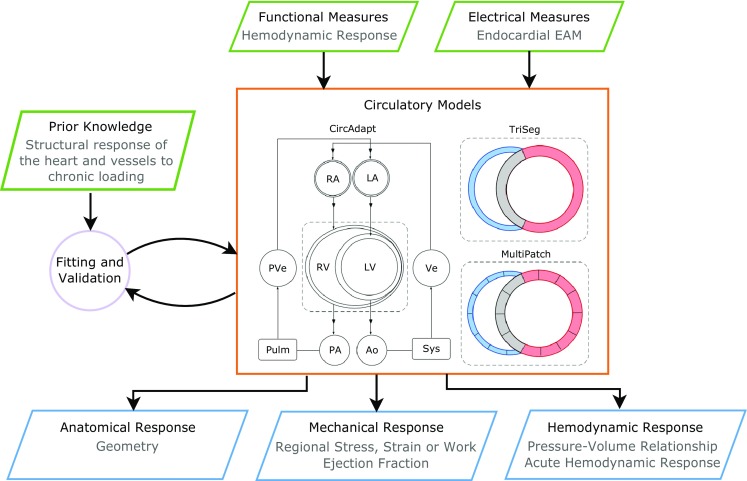
A schematic representation of the CircAdapt model together with the TriSeg and the MultiPatch models as its extensions is given in the orange box. Known physiological adaptation rules are used to adapt geometry parameters with the aim to match clinical measurements for mean systemic pressure and flow given in input to the model. The model gives in output hemodynamic and anatomical response in the form of geometry parameters as a consequence of adaptation, and pressure-volume relationships. When extended to the MultiPatch model, local electrical activation is needed as an additional input to provide information about stress, strain, and work within ventricular walls

The increased speed of these simulations does have limitations. Lumped-parameter models, together with the loss of spatial information, require additional parameters for the circulatory system that need to be estimated. This issue has been partially addressed by Arts et al. with the CircAdapt model [65]. Figure 5 gives a schematic representation of CircAdapt, which includes cardiac chambers linked to the systemic and the pulmonary circulation through cardiac valves. Geometry parameters for the components simulated by the model are adapted on the base of known physiological adaptation mechanisms to match measurements of systemic pressure and flow and, at the same time, to take into account such compensatory mechanisms. This framework relies on efficient simulations, with less parameters to estimate, and enables the simulation of a wide range of physiological and pathophysiological conditions [65]. This allows quantifying anatomical response, macroscopic hemodynamics as pressure-volume relationship, and clinical indices for pumping efficiency as ejection fraction and cardiac output.

Despite its high degree of flexibility, the original CircAdapt model is unable to account for intraventricular interaction, which plays an important role in HF and CRT, as the pump function of one ventricle is directly affected by the function of the other [142–145]. The TriSeg model was thus designed as an extension of the CircAdapt model to account for heterogeneity of electrical activation and wall tension of the three ventricular wall segments (left and right ventricular free walls and intraventricular septum) [66], as shown in Fig. 5. This framework was also used to simulate the effect of LBBB on ventricular pump function [146]. Results of this study agree with clinical observations on asynchronous hearts, with additional insight into LBBB consequences on other components of the circulatory system. The model is indeed able to predict functional mitral valve regurgitation as a direct consequence of LBBB, consistent with clinical observations [147].

In a further refinement to provide information about local deformation, the TriSeg model was further extended in the MultiPatch model [64]. The three ventricular wall segments were divided into an arbitrary number of patches assigned with an individual activation time from patient-specific measurements derived from endocardial EAM, allowing the model to simulate electrical activation heterogeneities and consequent local changes in the distribution of stress, strain, and work within a single wall. The MultiPatch model was used to simulate CRT delivery by means of LV and BiV pacing [148]. In this study, parameters of the model were tuned to match electrical activation maps measured in LBBB canine and HF patients’ hearts. Simulations showed that even though electrical activations consequent to LV and BiV pacing protocols are different, both pacing strategies lead to similarly improved cardiac pump function. This was explained by the similar increase in myocardial work, which was found to be linearly related to LV systolic function. The MultiPatch model also provides information about local deformations within the ventricular walls in agreement with animal data and tagged-MRI strain patterns. As already mentioned, the structure of the MultiPatch model allows to simulate heterogeneity of electrical activation as well as mechanical heterogeneity within a single ventricular wall segment [11]. Furthermore, each patch can be assigned with an individual activation time together with individual mechanical properties to simulate low contractility and stiff regions such as scar. Simulations of different LV pacing lead position in both ischemic and non-ischemic HF confirmed results reported in previous clinical studies [149, 150], with novel insight into the physiology at the base of these findings [11].

The CircAdapt model and its extensions described so far simulate macroscopic adaptation of cardiac chambers, with a limited representation of microscopic dynamics. When dealing with HF and CRT, the understanding of microscopic compensatory mechanisms may be important. The modular structure of CircAdapt makes it relatively easy to couple it with models for microscopic electrical activation and sarcomere contraction [151]. By coupling the TriSeg model with models for cellular excitation and sarcomere contraction for ventricular myocytes, Kuijpers et al. analyzed how microscopic mechanisms affect macroscopic cardiac function. The efficiency of the simulations offered by such framework allowed to simulate the dynamics of interest for several heart beats, thus replicating what happens in case of both acute and sustained LBBB, together with CRT delivery [151].

In summary, the CircAdapt model constitutes a valuable alternative to computationally intense three-dimensional models. Its modular structure makes CircAdapt easy to couple with more detailed frameworks for the dynamics of interest needed to address a specific clinical problem. The TriSeg and the MultiPatch model as extensions of CircAdapt partially overcome the lack of local information about electrical activation and mechanical deformation in ventricular walls, making these frameworks more suitable for LBBB and CRT simulations. The efficient simulations offered by CircAdapt also lead to a fast clinical translation compared to three-dimensional models for cardiac electromechanics. In future work, the coupling of these two modeling frameworks could prove invaluable in modeling changes in LBBB and reverse remodeling due to CRT in HF patients.

## Conclusion

In this review, we have discussed cardiac computer models used to investigate cardiac dysfunction, especially focusing on LBBB and CRT. Electrophysiology, electromechanical, and circulatory models have been used to identify the optimal pacing location and intracardiac timings to improve response to CRT, investigate the cardiac substrate (endocardial layers, scar burden or location) to provide insight into the pathologies in the heart that cause the electrical and mechanical dyssynchrony in LBBB and the response due to CRT, as well as to study new technologies such as multisite pacing or endocardial LV pacing.

The electromechanical models discussed in this review require extensive clinical information, oftentimes including invasive measurements. Future work requires the development of pipelines that allow for the autogeneration of models and parameters from non-invasive data, as well as methods to reduce the computational costs or improvements in the parallel scalability of the simulations.

Patient-specific models for CRT are often very complex with many free parameters. At the same time the large patient variability in the CRT patient populations motivates personalizing model parameters to each individual patient. However, efficiently and uniquely constraining model parameters remains a significant challenge in model creation. The inability to uniquely constrain parameters using available clinical data will in some cases limit the predictive ability of the model. However, in biological systems, not all parameters will have equal impact on all model predictions [152], allowing some parameters to be set at representative values, without compromising the models predictive capacity. Further, when models fail to make accurate predictions, we can identify important parameters that were not well constrained by available measurements. We can then use this information to identify measurements that need to be made to achieve better predictions for future patients. Finally, as models move from research techniques to clinical tools, there will need to be a greater emphasis placed on uncertainty quantification so that the effect of unknown or poorly constrained parameters on model predictions can be included in estimated confidence intervals that can in turn guide a clinician in the reliability of the model predictions.

This is especially true in light of the heterogeneous patient population within a standard CRT cohort, where questions remain unanswered regarding the accuracy and reliability of model predictions. The uncertainties inherent in the models due to reliability and robustness of data measurements (such as AHR) and modeling assumptions need to be addressed upfront when presenting biophysical models of the heart to clinicians. Computational modeling offer an additional tool to make clinical decisions; however, diagnostic and treatment decisions based on model simulations need to be made with the full knowledge of the errors and confidence in the models.

The current state of the computational modeling of LBBB and CRT patients rely on expertise in image processing, numerical analysis, mesh generation, and cardiac electrophysiology, mechanics and circulatory response which presents another barrier to clinical translation. The development of user-friendly tools that allow the non-expert model to model the electrical, functional, and anatomical response of the patient to CRT within a clinically useful timeframe remains a challenge for the community. The development of these tools requires close collaboration and feedback from clinical stakeholders.

To date, clinical translation of biophysical tools to model dyssynchrony and CRT response remain out of reach with current frameworks for creating and parameterizing patient-specific models requiring (1) substantial amount of information and (2) large amount of computational resources to simulate a single heartbeat. In addition, the robustness and accuracy of the measurements as well as the resulting model simulations/predictions need to be demonstrated.

Once these challenges have been met, dyssynchronous heart models can be used to: examine interventions in-silico; aid in clinical decisions of disease prognosis and prospective treatment plans; predict the long-term response to CRT for further classification of patients who would/would not benefit from CRT, thus reducing the need for unnecessary procedures impacting the health of patients; as well as provide a testing ground for emerging technologies.

## Abbreviations

CRT: (Cardiac Resynchronization Therapy)
LBBB: (Left bundle branch block)
DT-MRI: (Diffusion tensor magnetic resonance imaging)
MRI: (Magnetic resonance imaging)
VVD: (Interventricular delay)
AVD: (Atrioventricular delay)
Purkinje System: (PS)
ECC: (Excitation-contraction coupling)
MEF: (Mechano-electric feedback)
MSP: (Multisite pacing)
